# Abnormalities of retinal function in type 2 diabetes mellitus patients without clinical diabetic retinopathy detected by multifocal electroretinogram

**DOI:** 10.1186/s12886-024-03335-7

**Published:** 2024-02-15

**Authors:** Rong-Rong Li, Yang Yang, Meng-Ge Zhang, Jie Wang, Hong Chen, Shan Liu, Hui Miao, Yun-Chang Wang

**Affiliations:** 1 Hebei Provincial Key Laboratory of Ophthalmology, Hebei Eye Hospital, Hebei Provincial Clinical Research Center for Eye Diseases, NO. 399 quan bei dong da jie, 054001 Xingtai, Hebei Province China; 2 Beijing Yiran Changwu Cultural Creative Co., Ltd, Beijing, China

**Keywords:** Diabetic retinopathy, Multifocal electroretinogram, Type 2 diabetes mellitus

## Abstract

**Objective:**

To study the changes of retinal function in type 2 diabetes mellitus(DM) patients without apparently diabetic retinopathy via multifocal electroretinogram. Methods: Thirty-six type 2 DM patients (72 eyes) without visible diabetic retinopathy were selected as the experimental group, and thirty-five healthy subjects (70 eyes) were selected as the control group. All subjects were underwent multifocal electroretinogram (mf- ERG).

**Results:**

Compared with the control group, the implicit time delay of the P1 wave in the first ring, third ring, fourth ring, and fifth ring of the experimental group was significant (t = -3.154, *p* = 0.004, t = -8.21, *p* = 0.000, t = -3.067, *p* = 0.004, t = -4.443, *p* = 0.000, respectively). The implicit time of the N1 wave in the fourth- and fifth-ring were also significantly delayed compared with the control group (t = -3.549, *p* = 0.001, t = 2.961, *p* = 0.005, respectively). Compared with the control group, the implicit time of the P1 wave and N1 wave in the temporal region of the experimental group were delayed (t = -2.148, *p* = 0.037, t = -2.834, *p* = 0.007, respectively). There were no significant difference between the experimental group and the control group of the temporal area in the amplitude density of P1 wave, N1 wave. There was no difference in the implicit time and amplitude density of the N1 and P1 waves in the nasal region between the experimental group and the control group. The multifocal electroretinogram complex parameters showed better specificity and sensitivity in the diagnosis of diabetic retinopathy.

**Conclusion:**

The multifocal electroretinogram can detect abnormal changes in the retina of type 2 DM patients without visible diabetic retinopathy. The multifocal electroretinogram complex parameter is a potential indicator for the early diagnosis of diabetic retinopathy.

## Introduction

Diabetic retinopathy(DR) is the microvascular disease of DM in the eye, with a blindness rate that remains the highest among all eye diseases in China [1]. Once visible retinal lesions occur, impairments in both neural and vascular structures are often irreversible; thus, early intervention for DM patients without obvious fundus changes becomes particularly important.

As a non-invasive examination, multifocal electroretinography (mf-ERG) has been widely used to evaluate the retinal function of the posterior pole, which is composed of N1,P1andN2 waves. It has been documented that the primary generators of mf- ERG are on and off bipolar cells [2], with contributions mediated by L-, M- and S-cone mechanisms [3].Diabetic retinopathy is largely caused by defects of retinal capillaries in the inner nuclear layer, where the cell bodies of the bipolar cells are located. Thus, mfERG is well suited to the study of the diabetic retina [4].It has also been reported that the delay of implicit time (IT) of N1 and P1 wave indicates the damage of neurons which are shown before microvascular injuried, which can be used as a predictor of the occurrence of diabetic retinopathy [5–6]. Therefore, we focus on the variations in implicit time and amplitude density (AD) of N1 and P1 wave of DM patients for the early intervention of diabetic retinopathy.

## Data and methods

### General data

Thirty-six DMs (72 eyes) without obvious diabetes retinopathy [7] (Early Treatment of Diabetic Retinopathy Study [ ETDRS] level < 20,diabetic retinopathy absent) were included in the experimental group, and thirty-five health controls (70 eyes) were included in the control group. All subjects were underwent slit lamp anterior segment examination, indirect ophthalmoscope fundus examination after mydriasis, non-contact intraocular pressure examination, and fundus photography. The best corrected visual acuity (BCVA) was above 16/20, and there was no microaneurysm or exudation in the fundus picture. Other fundus abnormalities associated with glaucoma, high myopia, macular disease, venous occlusion, etc., were excluded. All subjects and their families were informed of the purpose and risk of the experiment, and they all agreed and signed an informed consent form, which was approved by the Ethics Committee of Hebei Eye Hospital.

### Multifocal electroretinogram

**Fig. 1:**
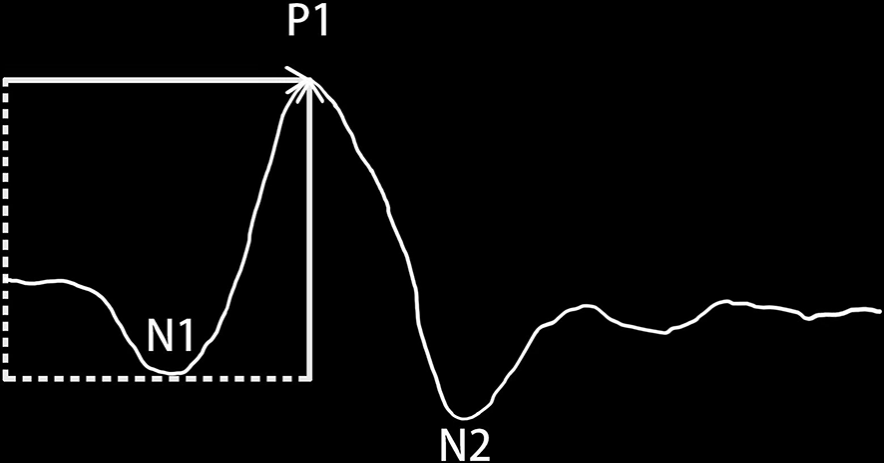
Diagram of a mfERG response to show the designation of the major features of the waveform. The arrows show the trough-to-peak amplitude (vertical arrow) and the implicit time. (horizontal arrow) of the basic mfERG measures of amplitude and timing [2]

**Fig. 2:**
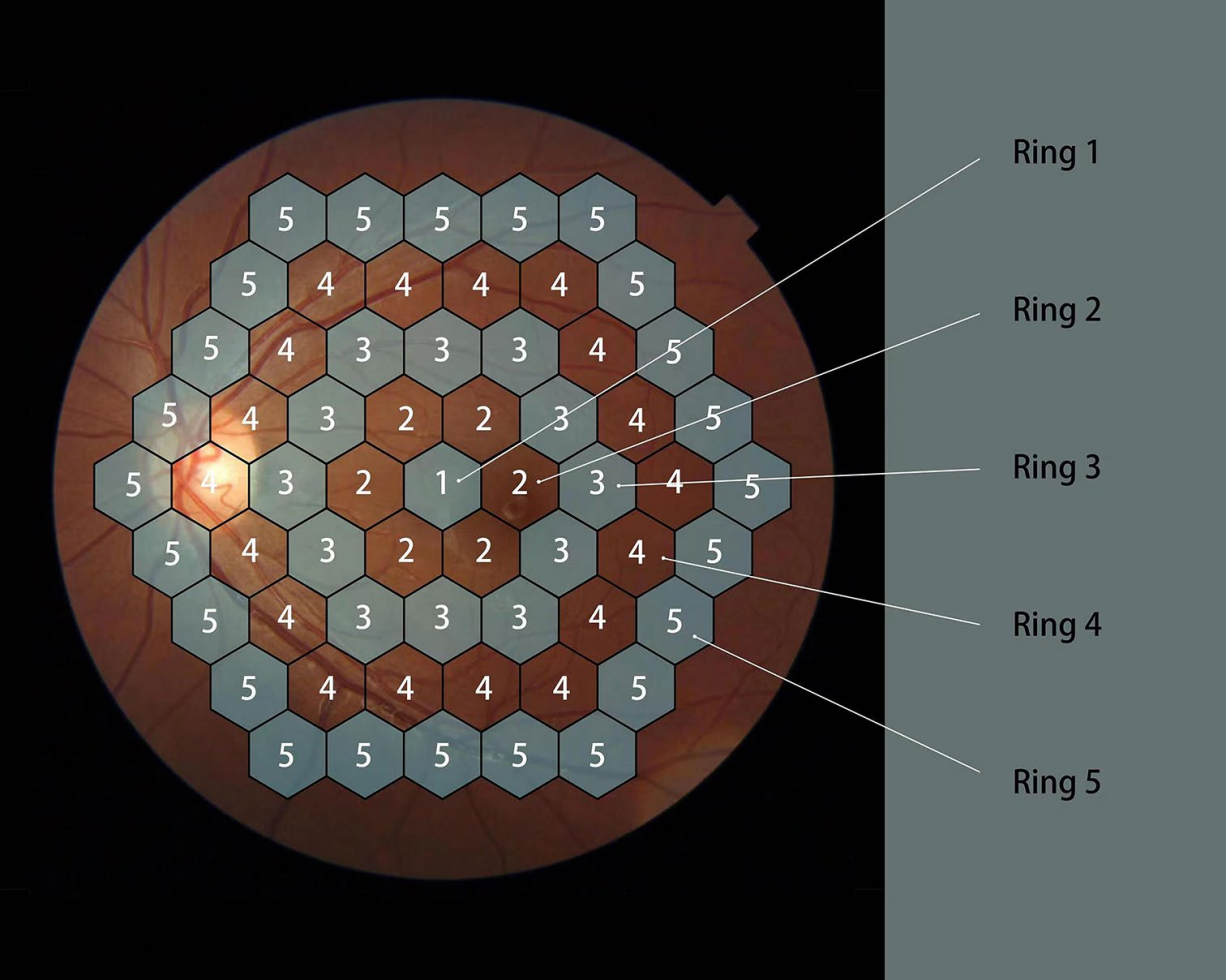
Image shows a fundus photo of the retina with five rings regions where the mfERG can distinguish the abnormalities of local retinal in posterior

According to ISCEV(International Society for Clinical,Electrophysiology of Vision) standard [2], Germany Roland RETI-Port/Scan 21 multifocal visual electrophysiology examination system was used. The mfERG was conducted in photopic condition with the pupil of the study eye dilated and was recorded using corneal contact lens electrodes. The distance from the chinrest to the stimulation screen was 28 cm and correction lenses (+ 3 diopter) was added to patient’s own refractive errors. The stimulations were displayed on a cathode ray tube (CRT) 19” monitor with a luminance at 220 cd/m2. The study eye was stimulated with 8 runs of 61 hexagonal elements, covering 54° of the retina (27°radius from fixation point) [2, 9], these stimulation units increased with the increase of eccentricity. The outcomes of mfERG were IT and AD of the first order kernel from the first ring to the fifth ring. The IT and AD of nasal and temporal N1 and P1 waves were recorded in four quadrants. The definition of IT and AD of the waveform was shown below in Fig. 1. the correspondence of fundus with five rings of mf- ERG was shown in Fig. 2.

## Statistical analysis

SPSS23.0 software was used for statistical analysis. The data were expressed in the form *x* ± sd. The comparison between the two groups was conducted by independent sample t-test. Mann–Whitney U test was used for the data which were not conformed to the normal distribution. The categorical variables were expressed in the form of percentage, and a chi-square test was used for comparison between groups. Pearson correlation analysis was used to assess the relationship between variables. When using a receiver operator characteristic (ROC) to analyze the implicit time, the probability of developing diabetes retinopathy is predicted according to the area under the curve (AUC). *P* < 0.05 indicates that the difference is statistically significant.

## Results

### General data analysis

The general data analysis of patients is shown in Table 1. There was no statistically significant difference in the distribution of all subjects in terms of age and sex (*P* > 0.05). The duration of diabetes in the experimental group was 7.06 ± 0.83 years, and there was no statistically significant difference in the BCVA and intraocular pressure between the two groups. The glycosylated hemoglobin in the experimental group was 5.77 ± 0.49, which was significantly higher than that in the control group (4.90 ± 0.46), and the difference was statistically significant (*P* < 0.05).

**Table 1:** Summary of general data

		Control group (CP)	Experimental (EP)	group p
Age (year)		65.37 ± 3.16	64.56 ± 3.19	0.469
Gender (male/female)		16/ 19	15/21	0.767
Course of diabetes (year)			7.06 ± 0.83	
HbA1C (%)		4.90 ± 0.46	5.77 ± 0.49	0.000
BCVA		4.98 ± 0.04	5.02 ± 0.35	0.642
Intraocular (mmHg) pressure		14.73 ± 1.53	14.19 ± 1.91	0.296

### Analysis of implicit time and amplitude density of N1 and P1 waves from the first ring to the fifth ring

There was no significant difference in the AD of N1 and P1 waves between the experimental group and the control group (*P* > 0.05). The IT of P1 waves of the first, third, fourth, and fifth rings in the experimental group was delayed compared with the control group, and the difference was significant (*t* = -3.154, *p* = 0.004, *t* = -8.21, *p* = 0.000, *t* = -3.067, *p* = 0.004, *t* = -4.443, *p* = 0.000, respectively). The IT of N1 waves of the fourth and fifth ring in the experimental group was significantly delayed compared with that in the control group (*t* = -3.549, *p* = 0.001, *t* = 2.961, *p* = 0.005, respectively), which were shown in the Table 2.

**Table 2:** Summary of implicit time and amplitude density of N1 and P1 waves from the first ring to the fifth ring

	CP	EP	t	*p*
AD of N1 in ring 1(nv/deg2 )	0.82 ± 0.16	0.74 ± 0.15	1.813	0.076
AD of N1 in ring 2(nv/deg2 )	0.36 ± 0.07	0.35 ± 0.07	0.524	0.603
AD of N1 in ring 3(nv/deg2 )	0.24 ± 0.04	0.23 ± 0.03	0.856	0.396
AD of N1 in ring 4(nv/deg2 )	0.23 ± 0.03	0.23 ± 0.05	-0.464	0.645
AD of N1 in ring 5(nv/deg2 )	0.18 ± 0.08	0.17 ± 0.04	0.540	0.593
IT of N1 in ring 1(ms)	19.12 ± 3.05	18.70 ± 4.53	0.373	0.711
IT of N1 in ring 2(ms)	22.36 ± 3.70	23.39 ± 3.86	-0.941	0.351
IT of N1 in ring 3(ms)	20.32 ± 2.79	18.39 ± 3.98	1.948	0.058
IT of N1 in ring 4(ms)	20.77 ± 4.43	24.24 ± 1.83	-3.549	0.001
IT of N1 in ring 5(ms)	25.41 ± 2.82	22.53 ± 3.86	2.961	0.005
AD of P1 in ring 1(nv/deg2 )	122.45 ± 11.76	115.32 ± 13.48	1.953	0.057
AD of P1 in ring 2(nv/deg2 )	39.27 ± 9.69	37.32 ± 8.58	0.737	0.465
AD of P1 in ring 3(nv/deg2 )	18.73 ± 2.46	18.75 ± 3.28	-0.028	0.977
AD of P1 in ring 4(nv/deg2 )	14.25 ± 3.46	12.97 ± 3.34	1.306	0.198
AD of P1 in ring 5(nv/deg2 )	8.76 ± 1.54	8.20 ± 1.79	1.176	0.246
IT of P1 in ring 1(ms)	45.81 ± 3.67	48.37 ± 1.55	-3.154	0.004
IT of P1 in ring 2(ms)	44.05 ± 2.06	44.92 ± 2.09	− 1.449	0.154
IT of P1 in ring 3(ms)	41.93 ± 1.55	45.45 ± 1.19	-8.821	0.000
IT of P1 in ring 4(ms)	44.14 ± 1.66	45.85 ± 2.17	-3.067	0.004
IT of P1 in ring 5(ms)	39.40 ± 1.95	41.53 ± 1.33	-4.443	0.000

### Analysis of implicit time and amplitude density of N1 wave and P1 wave in the nasal and temporal regions

**Fig. 3:**
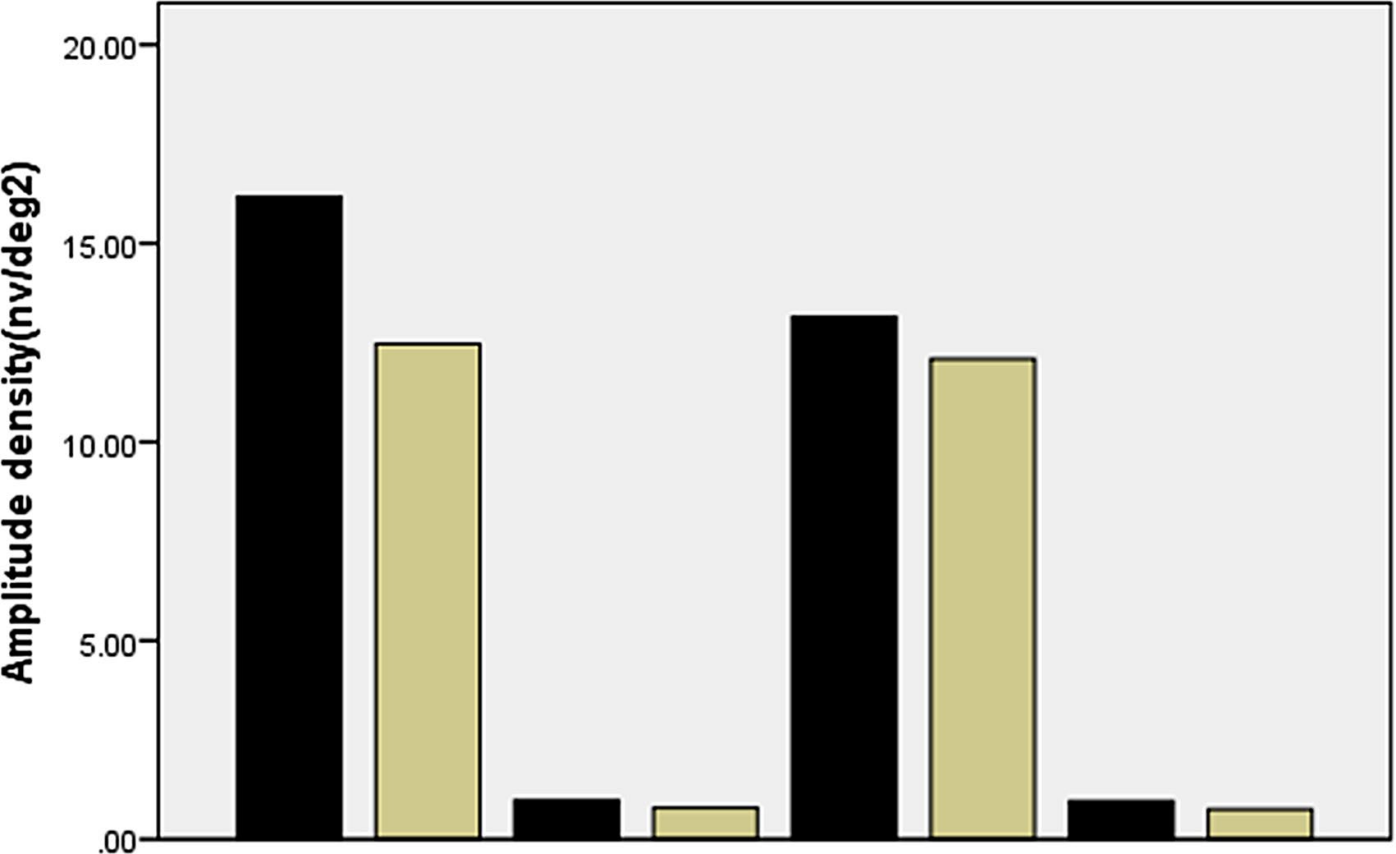
Comparision of AD (amplitude density) of N1 and P1 wave of nasal and temporal area of CP and EP

**Fig. 4:**
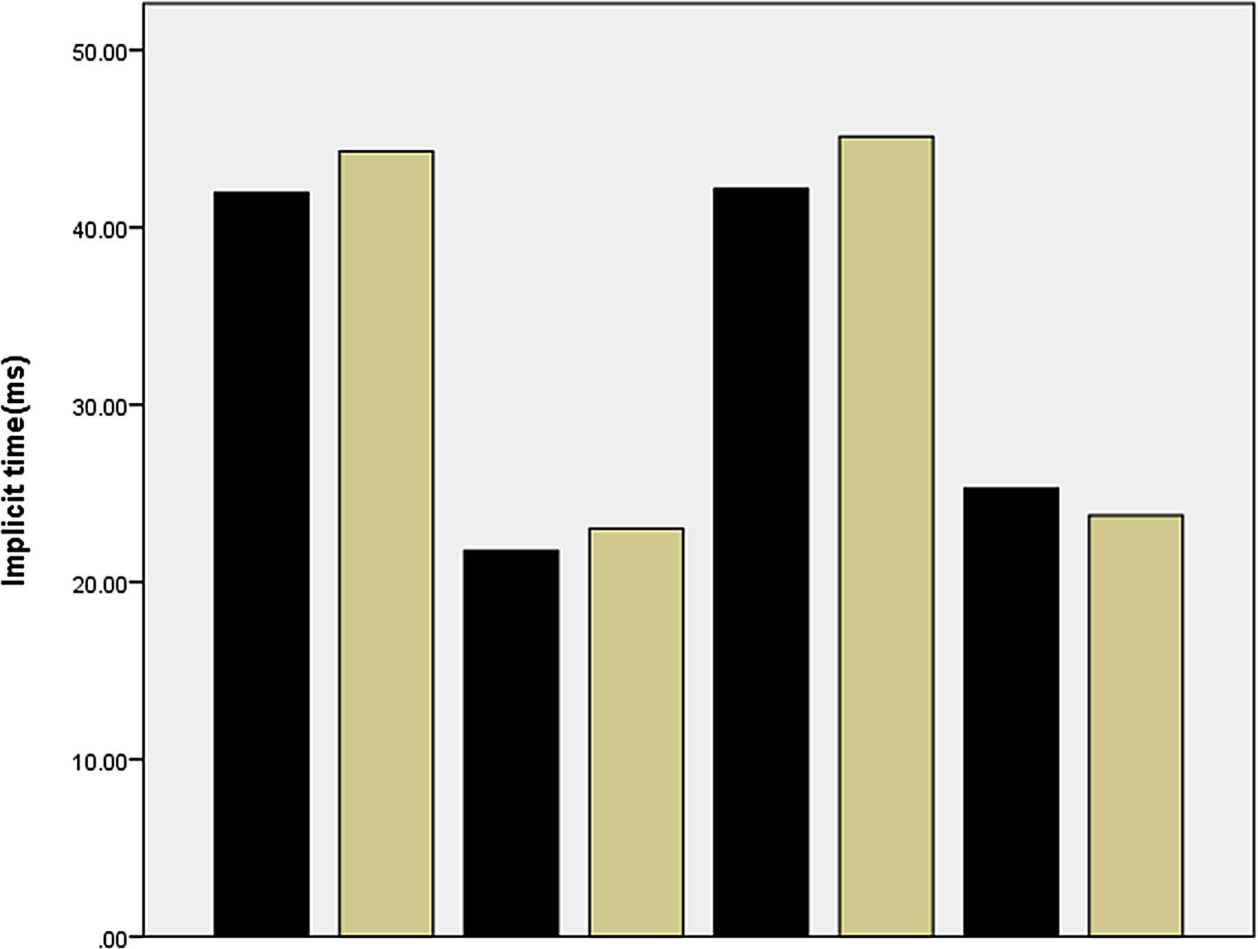
Comparision of IT (implicit time) of N1 and P1 wave of nasal and temporal area of CP and EP

Compared with the control group, the IT of the P1 wave and N1 wave in the temporal region of the experimental group were delayed (t = -2.148, *p* = 0.037, t = -2.834, *p* = 0.007, respectively). There were no significant difference between the experimental group and the control group of the temporal area in the amplitude density of P1 wave, N1 wave. No differences were shown between the two groups in the parameters of nasal area (the implicit time and amplitude density of N1 wave, P1 wave) in Figs. 3 and 4.

### Results of implicit time analysis of the subject’s working curve

**Fig. 5:**
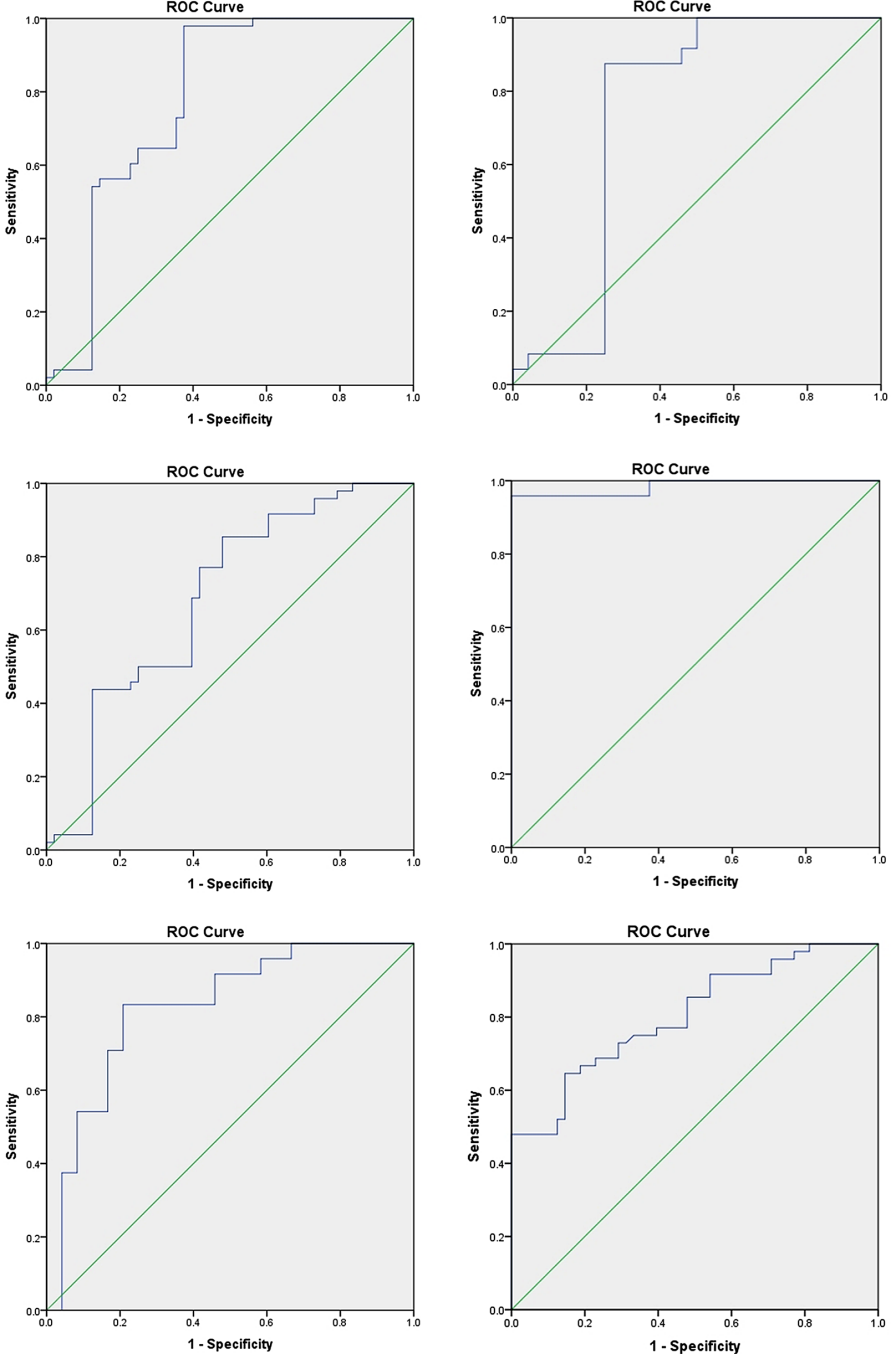
AUC of single and composite parameter. **(a)**–**(c)** shows the probability of diabetes retinopathy predicted by a single parameter, and **(d)**–**(f)** shows the probability of diabetes retinopathy predicted by a composite parameter. IT: implicit time; R1: ring 1; R3: ring 3; R5: ring 5

The area under the ROC curve of the first, third, and fifth rings of the experimental group were 0.779, 0.740, and 0.684, respectively. After the composite analysis of the implicit time of the first ring and third ring, third ring and fifth ring, as well as first ring and fifth ring, the area under the ROC curve were 0.984, 0.826, and 0.804, respectively, which were shown to have greater significance for the diagnosis of diabetes retinopathy in Fig. 5.

## Discussion

mfERG studies have indicated that reduced amplitudes and/or response delays both in areas in DM patients with and without diabetic retinopathy.The magnitude of mfERG IT delays has been shown to correlate with the severity of retinopathy and the locations of abnormal IT correlate spatially with anatomic abnormalities [8–9]. These areas may precede the onset of new structural signs of diabetic retinopathy. The loci of retina that appeared clinically normal but had functional abnormalities remained mostly stable; however, after a 1-year follow-up, they were more at risk for the development of microaneurysms compared to zones with a normal baseline implicit time[10].

Perfusion defects due to choriocapillaris degeneration may result in compromised local metabolism affecting the function of the mfERG generators leading to delayed neuronal conduction and be responsible for IT abnormalities. Amplitude reduction may indicate a more advanced condition where portions of the retina do not generate mfERG due to cell loss [11].

There are debating points on the change of amplitude. Due to the increase of retinal blood flow in diabetes patients and the heterogeneity of its distribution [12– 14], some studies [15] have concluded that the amplitude of mfERG’s first and second order responses increase. On the contrary, other studies [16] have suggested that the amplitude of early diabetes patients is lower than those without diabetes. Our study showed that the P1 wave AD of the first to fifth ring was lower than in the control group, but there was no statistical difference, which is consistent with a previous study [17]. The decrease of AD was not proportional to the delay of IT.AD, though reduced in DR, have no such correspondence with the presence of retinal lesions. This may be due to larger intersubject variability in AD rather than IT in normal subjects [10].

In this study, we found a significant delay of IT of the P1 and N1 waves in some areas of the retina in the experimental group respect to the control group. This might be due to the occlusion of the capillary network in the inner nuclear layer at early stage of DM, which led to ischemia, hypoxia and an undetectable non-perfusion area [18]. Nevertheless, there were still no instances of apparently retinopathy, such as microaneurysms and hard exudates.

Bearse and coworkers [19] found decreased uneven retinal distribution of the IT delays in patients with no DR compared to patients with non-proliferative DR. Abnormalities were more often found in the inferior retina, probably due to its greater ischemic susceptibility [20]. However, in our study, the temporal region was shown more sensitive to hyperglycemic injury than the nasal region, which may be because the temporal retinal vessels have poor expansion reserve capacity under the conditions of ischemia and hypoxia and thus are more intolerant to hypoxia [21–23]. Another reason may be that more cone cells and ganglion cells are distributed in the nasal and macular regions [23], while mfERG simply records the functions of bipolar cells and the inner segments of photoreceptors.

Our research innovatively proposes a method for analyzing the two separated rings together in order to reflect the retinal function abnormality of diabetes patients who ignore retinopathy at an early stage. The composite ROC curve showed higher specificity and sensitivity in the diagnosis of retinal abnormalities in early diabetes patients, and its area under the curve was larger than that of a single ring. Among them, the area under the compound curve of the first ring and third ring was the largest, revealing that the abnormality of the posterior pole was the main source of the whole retinal function defect when there was no visible retinal damage at an early stage.

In general, the function of the retina has been abnormal for a significant period before diabetes patients have visual retinopathy. For diabetes retinopathy, age-related macular degeneration and other major local retinal (posterior pole) diseases, mfERG can effectively identify early abnormalities. Ischemia, hypoxia and local metabolic changes may be the main reasons for the delay of IT in early diabetes patients. This study confirmed that mfERG can more sensitively reflect the abnormality of retinal function and can be used to evaluate the retinal function of early diabetes patients without visible retinopathy.

## Data Availability

The datasets used and/or analysed during the current study are available from the corresponding author on reasonable request.
